# PDI augments kainic acid-induced seizure activity and neuronal death by inhibiting PP2A-GluA2-PICK1-mediated AMPA receptor internalization in the mouse hippocampus

**DOI:** 10.1038/s41598-023-41014-7

**Published:** 2023-08-25

**Authors:** Duk-Shin Lee, Tae-Hyun Kim, Hana Park, Ji-Eun Kim

**Affiliations:** 1 Department of Anatomy and Neurobiology, College of Medicine, Hallym University, Chuncheon, Kangwon-do 24252 South Korea; 2 Institute of Epilepsy Research, College of Medicine, Hallym University, Chuncheon, 24252 South Korea

**Keywords:** Cell death in the nervous system, Diseases of the nervous system

## Abstract

Protein disulfide isomerase (PDI) is a redox-active enzyme and also serves as a nitric oxide donor causing *S*-nitrosylation of cysteine residues in various proteins. Although PDI knockdown reduces *α*-amino-3-hydroxy-5-methylisoxazole-4-propionic acid receptor (AMPAR)-mediated neuronal activity, the underlying mechanisms are largely unknown. In the present study, we found that under physiological condition PDI knockdown increased CaMKII activity (phosphorylation) in the mouse hippocampus. However, PDI siRNA inhibited protein phosphatase (PP) 2A-mediated GluA2 S880 dephosphorylation by increasing PP2A oxidation, independent of *S*-nitrosylation. PDI siRNA also enhanced glutamate ionotropic receptor AMPA type subunit 1 (GluA1) S831 and GluA2 S880, but not GluA1 S845 and GluA2 Y869/Y873/Y876 phosphorylations, concomitant with the enhanced protein interacting with C kinase 1 (PICK1)-mediated AMPAR internalization. Furthermore, PDI knockdown attenuated seizure activity and neuronal damage in response to kainic acid (a non-desensitizing agonist of AMPAR). Therefore, these findings suggest that PDI may regulate surface AMPAR expression through PP2A-GluA2-PICK1 signaling pathway, and that PDI may be one of the therapeutic targets for epilepsy via AMPAR internalization without altering basal neurotransmission.

## Introduction

The *N*-methyl-*D*-aspartate receptor (NMDAR) is one of the major excitatory receptors, which contributes to regulation of neurotransmission, synaptic plasticity and neuronal damage. Although mechanistic regulations of NMDAR channel activity are basically relevant to the bindings of ligands (e.g. glutamate and NMDA) and modulators (e.g. glycine, D-serine and Mg^2+^), redox modulation of reactive disulfide bonds on NMDAR also affects the conformational changes in the NMDAR functionality. For instance, reducing agents (dithiothreitol (DTT), H_2_S, glutathione (GSH)) inducing thiolation of disulfide bonds (S–S → HS– + –SH) reinforce NMDAR-mediated currents, while oxidizing agents (e.g. 5–5′-dithiobis-2-nitrobenzoic acid (DTNB) and oxidized glutathione (GSSG)) leading to disulfide formation (HS– + –SH → S–S) inhibit them. Furthermore, *S*-nitrosylation (–SH → –SNO) of cysteine (C) residues reduces NMDAR functionality^1–3^. Although the oxidation of NMDAR redox sites decreases NMDAR-mediated currents by inhibiting channel openings, it cannot block physiological NMDAR functions including synaptic plasticity, such as inductions of long-term potentiation (LTP) and long-term depression (LTD)^2,4,5^. However, NMDAR redox inhibits seizure activity induced by kainic acid (KA), bicuculline, pilocarpine, pentylenetetrazol, NMDA and low Mg^2+^ concentration^5,6^. In spite of the absence of reducing agents, furthermore, seizure activity causes the reduction of NMDAR redox sites leading to the potentiation of NMDAR function even after the cessation of epileptiform discharges^5^. Thus, it is likely that unknown endogenous reducing factors would be involved in these phenomena.

Protein disulfide isomerase (PDI) is a redox-active enzyme, which catalyzes disulfide bonds formation, reduction or isomerization of various proteins in the lumen of the endoplasmic reticulum (ER), cytoplasm and cell surface^7^. Interestingly, PDI inhibition protects neurons from glutamate-induced oxidative cytotoxicity^8^. Similar to DTNB also known as PDI inhibitor^9^, PDI knockdown reduces the amounts of free thiols on NMDAR subunits (GluN1 and GluN2A) and attenuates acute seizure activity in response to pilocarpine and NMDA as well as spontaneous seizures in chronic epilepsy rats independent of *S*-nitrosylation, while it does not induce alterations in basal neurotransmission (paired-pulse response) and ER stress under physiological condition^6,10^. Thus, we have reported that PDI might be one of the reductive enzymes regulating the redox-mediated NMDAR activity.

On the other hand, activity-dependent alterations in synaptic strength are mediated by *α*-amino-3-hydroxy-5-methylisoxazole-4-propionic acid receptor (AMPAR) in response to NMDAR activity. Indeed, NMDAR-mediated Ca^2+^ entry, and the subsequent activations of Ca^2+^/calmodulin (CaM)-dependent kinase II (CaMKII) and protein kinase C (PKC) increase AMPAR channel conductance by increasing serine (S) 831 phosphorylation of glutamate ionotropic receptor AMPA type subunit 1 (GluA1). Furthermore, NMDAR activation enhances protein kinase A (PKA)-mediated GluA1 S845 phosphorylation, which regulates AMPAR peak response open probability and synaptic trafficking of AMPARs^11,12^. However, NMDAR activation also facilitates AMPAR internalization by enhancing PKC- and CaMKII-mediated GluA2 S880 phosphorylation^13,14^. On note, DTNB-induced NMDAR oxidation cannot affect AMPAR- and γ-aminobutyric acid receptor (GABAR)-mediated synaptic responses, although it attenuates seizure activity^15,16^. Therefore, it is likely that NMDAR redox would not involve the activity-dependent regulation of AMPAR functionality. However, we have found that PDI knockdown reduces the amplitude and frequency of neuronal discharges in response to AMPA as well as NMDA without altering GABAergic inhibitions^6,10^. These discrepancies would be consequences from properties of DTNB and PDI siRNA: DTNB oxidizes C residues on proteins exposed to the extracellular milieu, because of poor membrane permeability^17^. Unlike DTNB, PDI siRNA regulates the thiol modification of proteins in ER, cytosol and cell surface^6,10,18,19^. Furthermore, PDI serves as a nitric oxide (NO) donor causing *S*-nitrosylation of C residues that is a posttranslational protein modification^20,21^. Therefore, it is noteworthy exploring the underlying mechanisms of the blockade of AMPAR-mediated neuronal activity induced by PDI knockdown, which has been largely unknown.

Here, we found that under physiological condition PDI knockdown inhibited PKC, but increased CaMKII activity (phosphorylation) in the mouse hippocampus without affecting PKA activity. However, PDI siRNA enhanced GluA1 S831 and GluA2 S880, but not GluA1 S845 and GluA2 Y869/Y873/Y876 phosphorylations, and diminished surface AMPAR expression. These phenomena were relevant to the inhibition of protein phosphatase (PP) 2A by enhancing disulfide bond formation (oxidation), independent of *S*-nitrosylation. Furthermore, PDI knockdown attenuated seizure activity and neuronal damage in response to KA, a non-desensitizing agonist of AMPAR^22,23^, by rapidly increasing protein interacting with C kinase 1 (PICK1)-mediated AMPAR internalization. Therefore, these findings indicate that PDI may regulate surface AMPAR expression via PP2A-mediated GluA2 S880 dephosphorylation, and suggest that PDI may be one of the therapeutic targets for epilepsy via AMPAR internalization without altering basal neurotransmission.

## Results

### PDI knockdown increases GluA1 S831, but not S845, phosphorylation

**Figure 1:**
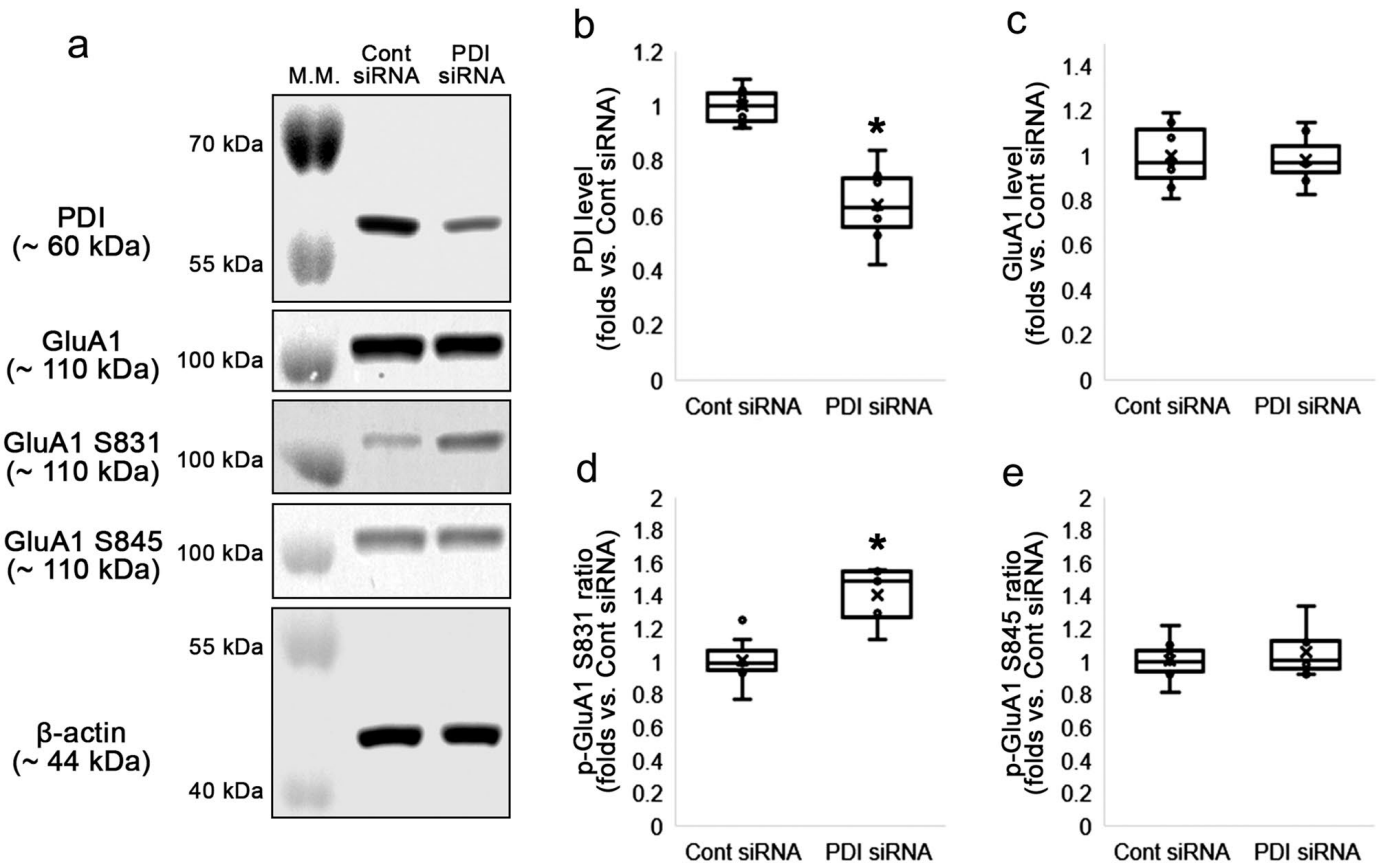
Effects of PDI knockdown on GluA1 phosphorylation in the hippocampus under physiological condition. As compared to control siRNA, PDI siRNA enhances GluA1 S831, but not S845, phosphorylation in the mouse hippocampus. (**a**) Representative Western blot images for PDI, GluA1, p-GluA1 S831 and p-GluA1 S845 levels. (**b**–**e**) Quantitative analyses of the effects of PDI siRNA on PDI (**b**), GluA1 (**c**), p-GluA1 S831 (**d**) and p-GluA1 S845 levels (**e**) based on the Western blot data (*p < 0.05 vs. control siRNA; *n* = 7, respectively; Mann–Whitney test).

First, we explored whether PDI knockdown affect GluA1 phosphorylations, since phosphorylation of AMPAR is a major mechanism for the regulation of receptor function and surface expression: GluA1 S831 and/or S845 phosphorylation promote surface AMPAR insertion and synaptic retention, and increase channel open-probability^11,24,25^. PDI siRNA decreased PDI protein level to 0.64-fold of control siRNA level in the mouse hippocampus (Z = 3.13, *p* = 0.002, *n* = 7, respectively, Mann–Whitney test; Fig. 1a–b, Supplementary Fig. 1). PDI siRNA did not affect GluA1 total protein level (Z = 0.064, *p* = 0.949, *n* = 7, respectively, Mann–Whitney test; Fig. 1a and c, Supplementary Fig. 1). However, PDI knockdown enhanced GluA1 S831 phosphorylation (p-GluA1 S831 level) to 1.37-fold of control siRNA level (Z = 3.003, *p* = 0.003, *n* = 7, respectively, Mann–Whitney test) and GluA1 S831 phosphorylation ratio (p-GluA1 S831 ratio) to 1.41-fold of control siRNA level (Z = 2.875, *p* = 0.004, *n* = 7, respectively, Mann–Whitney test; Fig. 1a and d, Supplementary Fig. 1). However, GluA1 S845 phosphorylation (p-GluA1 S845 level; Z = 0.576, *p* = 0.564, *n* = 7, respectively, Mann–Whitney test) and GluA1 S845 phosphorylation ratio (p-GluA1 S845 ratio; Z = 0.703, *p* = 0.482, *n* = 7, respectively, Mann–Whitney test; Fig. 1a and e, Supplementary Fig. 1) were unaffected by PDI knockdown. These findings indicate that PDI may negatively regulate GluA1 S831 phosphorylation without GluA1 S845 phosphorylation under physiological condition.

### PDI siRNA enables GluA2 S880 phosphorylation and reduces AMPAR surface expression under physiological condition

**Figure 2:**
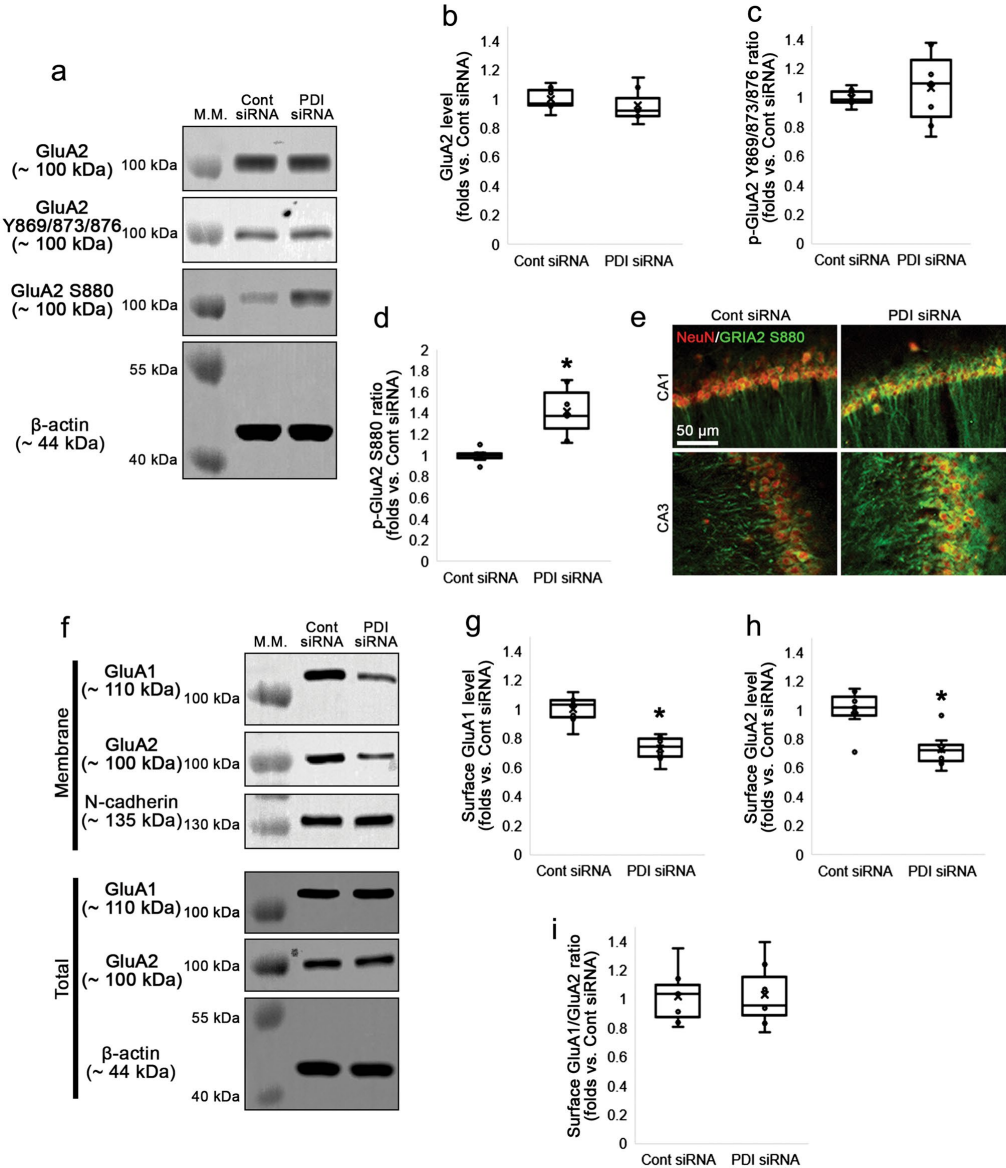
Effects of PDI knockdown on GluA2 phosphorylation and surface AMPAR expression in the hippocampus under physiological condition. As compared to control siRNA, PDI siRNA enhances GluA2 S880, but not Y869/873/876, phosphorylation in the mouse hippocampus. GluA2 S880 upregulations are observed in the dendrites and cell bodies of hippocampal neurons. However, PDI siRNA reduces surface expression of GluA1 and GluA2 without affecting surface GluA1/GluA2 ratio. (**a**) Representative Western blot images for GluA2, p-GluA2 Y869/873/876 and p-GluA2 S880 levels. (**b**–**d**) Quantitative analyses of the effects of PDI siRNA on GluA2 (**b**), p-GluA2 Y869/873/876 (**c**) and p-GluA2 S880 levels (**d**) based on the Western blot data (*p < 0.05 vs. control siRNA; *n* = 7, respectively; Mann–Whitney test). (**e**) Representative double immunostaining for NeuN and GluA2 S880 in CA1 and CA3 pyramidal neurons. (**f**) Representative Western blot images for surface expressions of GluA1 and GluA2. (**g**–**i**) Quantitative analyses of the effects of PDI siRNA on surface GluA1 (**g**) and GluA2 (**h**) levels and surface GluA1/GluA2 ratio (**i**) based on the Western blot data (*p < 0.05 vs. control siRNA; *n* = 7, respectively; Mann–Whitney test).

Next, we investigated whether PDI regulates GluA2 phosphorylation in the mouse hippocampus under physiological condition. To confirm this, we evaluated the effect of PDI knockdown on GluA2 phosphorylations and AMPAR surface expression. PDI siRNA did not alter GluA2 total protein level (Z = 1.217, *p* = 0.224, *n* = 7, respectively, Mann–Whitney test; Fig. 2a and b, Supplementary Fig. 2). PDI knockdown did not affect GluA2 Y869/Y873/Y876 phosphorylation (p-GluA2 Y869/873/876 level; Z = 0.192, *p* = 0.848, *n* = 7, respectively, Mann–Whitney test) and GluA2 Y869/Y873/Y876 phosphorylation ratio (p-GluA2 Y869/873/876 ratio; Z = 0.575, *p* = 0.565, *n* = 7, respectively, Mann–Whitney test; Fig. 2a and c, Supplementary Fig. 2). However, PDI siRNA enhanced GluA2 S880 phosphorylation (p-GluA2 S880 level) to 1.33-fold of control siRNA level (Z = 3.13, *p* = 0.002, *n* = 7, respectively, Mann–Whitney test). Thus, PDI knockdown elevated GluA2 S880 phosphorylation ratio (p-GluA2 S880 ratio) to 1.41-fold of control siRNA level (Z = 3.134, *p* = 0.002, *n* = 7, respectively, Mann–Whitney test; Fig. 2a and d, Supplementary Fig. 2). Double immunofluorescent studies revealed that GluA2 S880 signals were upregulated in the dendrites and cell bodies in hippocampal neurons (Fig. 2e). In addition, PDI siRNA decreased surface GluA1 (Z = 0.256, *p* = 0.798, *n* = 7, respectively, Mann–Whitney test) and GluA2 surface expressions (Z = 0.961, *p* = 0.336, *n* = 7, respectively, Mann–Whitney test), as compared to control siRNA (Fig. 2f-h, Supplementary Fig. 2). However, PDI siRNA did not alter surface GluA1/GluA2 ratio (Fig. 2f and i, Supplementary Fig. 2). GluA2 S880 phosphorylation facilitates AMPAR internalization^18,19^, while GluA1 S831 phosphorylation is not required for AMPAR trafficking^26,27^. Therefore, our findings indicate that PDI siRNA may diminish surface AMPAR expression by increasing p-GluA2 S880 level under physiological condition.

### PDI knockdown reduces PKC, but increases CaMKII activity

**Figure 3:**
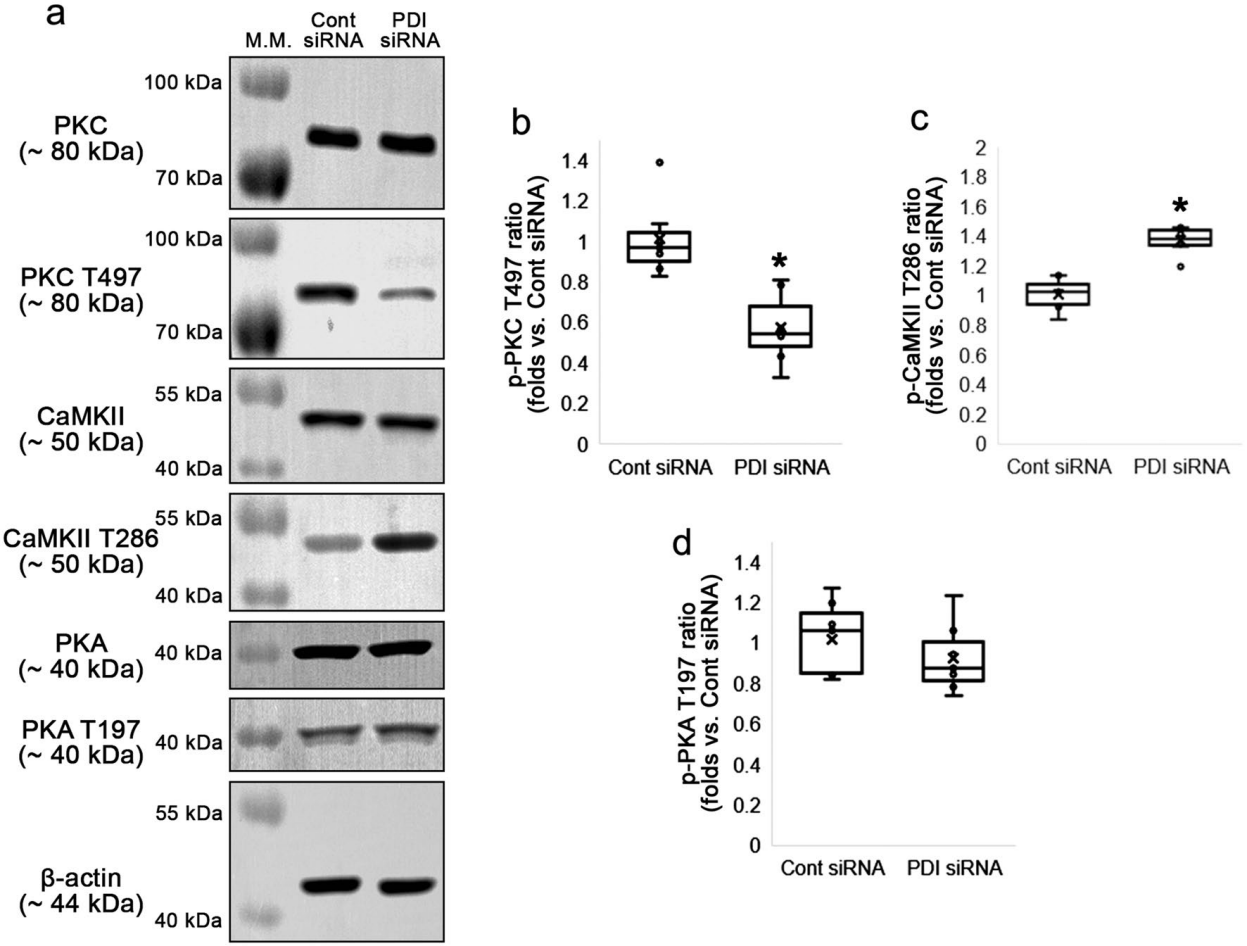
Effects of PDI knockdown on PKC, CaMKII and PKA phosphorylations in the hippocampus under physiological condition. As compared to control siRNA, PDI siRNA reduces PKC T497 phosphorylation, while it enhances CaMKII T286 phosphorylation. PDI knockdown does not affect PKA T197 phosphorylation in the mouse hippocampus. (**a**) Representative Western blot images for PKC, p-PKC T497, CaMKII, p-CaMKII T286, PKA and p-PKA T197 levels. (**b**–**d**) Quantitative analyses of the effects of PDI siRNA on p-PKC T497 (**b**), p-CaMKII T286 (**c**) and p-PKA T197 levels (**d**) based on the Western blot data (*p < 0.05 vs. control siRNA; *n* = 7, respectively; Mann–Whitney test).

Since both PKC and CaMKII phosphorylate GluA1 S831 and GluA2 S880 sites^12–14^, we explored whether PDI siRNA influences PKC and CaMKII activities (phosphorylations). Although PDI siRNA did not change PKC total protein level (Z = 0.064, *p* = 0.949, *n* = 7, respectively, Mann–Whitney test; Fig. 3a–b), it diminished PKC threonine (T) 497 phosphorylation (p-PKC T497 level) to 0.56-fold of control siRNA level (Z = 3.13, *p* = 0.002, *n* = 7, respectively, Mann–Whitney test) and PKC T497 phosphorylation ratio (p-PKC T497 ratio) to 0.57-fold of control siRNA level (Z = 3.13, *p* = 0.002, *n* = 7, respectively, Mann–Whitney test; Fig. 3a–b, Supplementary Fig. 3). In contrast to PKC, PDI knockdown increased CaMKII T286 phosphorylation (p-CaMKII T286 level) to 1.4-fold of control level (Z = 3.003, *p* = 0.003, *n* = 7, respectively, Mann–Whitney test) without affecting CaMKII protein level (Z = 0.450, *p* = 0.653, *n* = 7, respectively, Mann–Whitney test). Thus, CaMKII T286 phosphorylation ratio (p-CaMKII T286 ratio) was also increased to 1.37-fold of control level following PDI knockdown (Z = 3.13, *p* = 0.002, *n* = 7, respectively, Mann–Whitney test; Fig. 3a and c, Supplementary Fig. 3). Compatible with GluA1 S845 phosphorylation, total protein level of PKA (a kinase of GluA1 S845 site^11^) was unaltered by PDI siRNA (Z = 0.832, *p* = 0.405, *n* = 7, respectively, Mann–Whitney test). PKA T197 phosphorylation (p-PKA T197 level; Z = 0.705, *p* = 0.481, *n* = 7, respectively, Mann–Whitney test) and PKA T197 phosphorylation ratio (p-PKA T197 ratio; Z = 0.958, *p* = 0.338, *n* = 7, respectively, Mann–Whitney test; Fig. 3a and d, Supplementary Fig. 3) were also unchanged by PDI knockdown. These findings indicate that PDI siRNA may increase CaMKII-mediated GluA1 S831 and GluA2 S880 phosphorylation without altering PKA-mediated GluA1 S845 phosphorylation, under physiological condition.

### PDI knockdown reduces the amount of total thiols on PP2A, independent of *S*-nitrosylation

**Figure 4:**
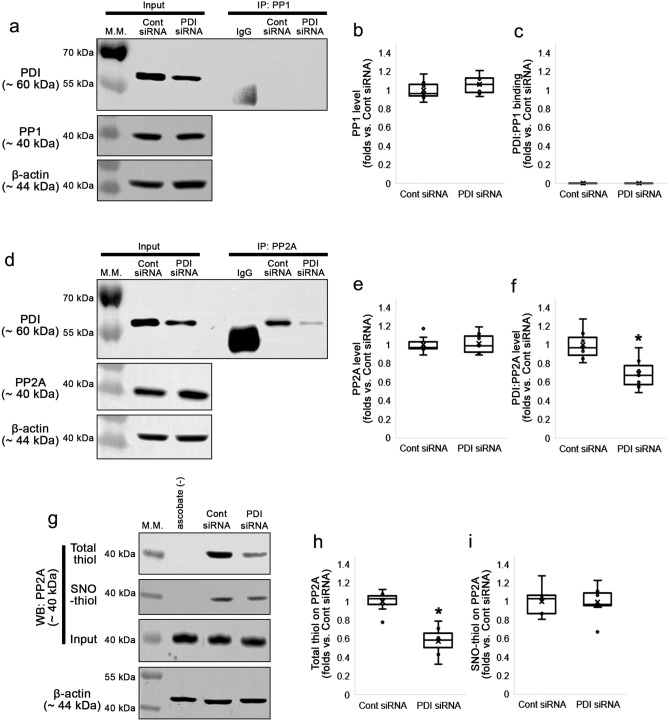
Effects of PDI knockdown on PDI bindings to PP1 and PP2A, and the thiolization and *S*-nitrosylation and on PP2A in the hippocampus under physiological condition. As compared to control siRNA, PDI siRNA reduces PDI:PP2A bindings without affecting PDI:PP1 bindings. In addition, PDI knockdown reduced the amount of total thiols on PP2A, while it does not affect that of SNO-thiols in the mouse hippocampus. (**a**) Representative Western blot images for the PDI:PP1 bindings. (**b**–**c**) Quantitative analyses of the effects of PDI siRNA on PP1 level (**b**) and PDI:PP1 binding (**c**) based on the Western blot data (*n* = 7, respectively). (**d**) Representative Western blot images for the PDI:PP2A bindings. (**e**–**f**) Quantitative analyses of the effects of PDI siRNA on PP2A level (**e**) and PDI:PP2A binding (**f**) based on the Western blot data (*p < 0.05 vs. control siRNA; *n* = 7, respectively; Mann–Whitney test). (**g**) Representative Western blot images for the thiolization and *S*-nitrosylation on PP2A. (**h**–**i**) Quantitative analyses of the effects of PDI siRNA on thiolization (**h**) and *S*-nitrosylation on PP2A (**i**) based on the Western blot data (*p < 0.05 vs. control siRNA; *n* = 7, respectively; Mann–Whitney test).

Since PDI has no kinase or phosphatase activity, how PDI knockdown enhanced CaMKII-mediated GluA1 S831 and GluA2 S880 phosphorylation was uncertain. CaMKII T286 autophosphorylation generates Ca^2+^-independent prolonged kinase activity, which is dephosphorylated by PP1 and PP2A^28,29^. GluA2 S880 site is also dephosphorylated by PP1 and PP2A, but not PP2B^30,31^. Interestingly, redox status or *S*-nitrosylation of cysteine (C) residues regulates phosphatase activity^32–34^. Given that PDI is a redox-active enzyme and a NO supplier^7,20,21^, it is likely that PDI siRNA may enhance CaMKII-mediated GluA1 S831 and GluA2 S880 phosphorylation by regulating redox and/or *S*-nitrosylation of PP1 and PP2A. In the present study, co-immunoprecipitation data showed PDI bound to PP2A, but not PP1, under physiological condition (Fig. 4a–f, Supplementary Fig. 4). PDI siRNA did not affect PP1 total protein level (Z = 1.091, *p* = 0.275, *n* = 7, respectively, Mann–Whitney test; Fig. 4a-b, Supplementary Fig. 4). Although PP2A total protein level was unaffected by PDI knockdown (Z = 0.128, *p* = 0.898, *n* = 7, respectively, Mann–Whitney test; Fig. 4d–e, Supplementary Fig. 4), PDI siRNA reduced PDI-PP2A binding (Z = 2.558, *p* = 0.011, *n* = 7, respectively, Mann–Whitney test; Fig. 4d and f, Supplementary Fig. 4). In addition, PDI siRNA decreased the amount of total thiol on PP2A to 0.58-fold of control siRNA level (Z = 3.003, *p* = 0.003, *n* = 7, respectively, Mann–Whitney test; Fig. 4g–h). The amount of SNO-thiol on PP2A was unaffected by PDI knockdown (Z = 0.192, *p* = 0.848, *n* = 7, respectively, Mann–Whitney test; Fig. 4g and i, Supplementary Fig. 4). These findings indicate that PDI siRNA may inhibit PP2A activity by oxidizing free thiols to disulfide bonds under physiological condition, independent of *S*-nitrosylation.

### PDI knockdown attenuates KA-induced seizure susceptibility

**Figure 5:**
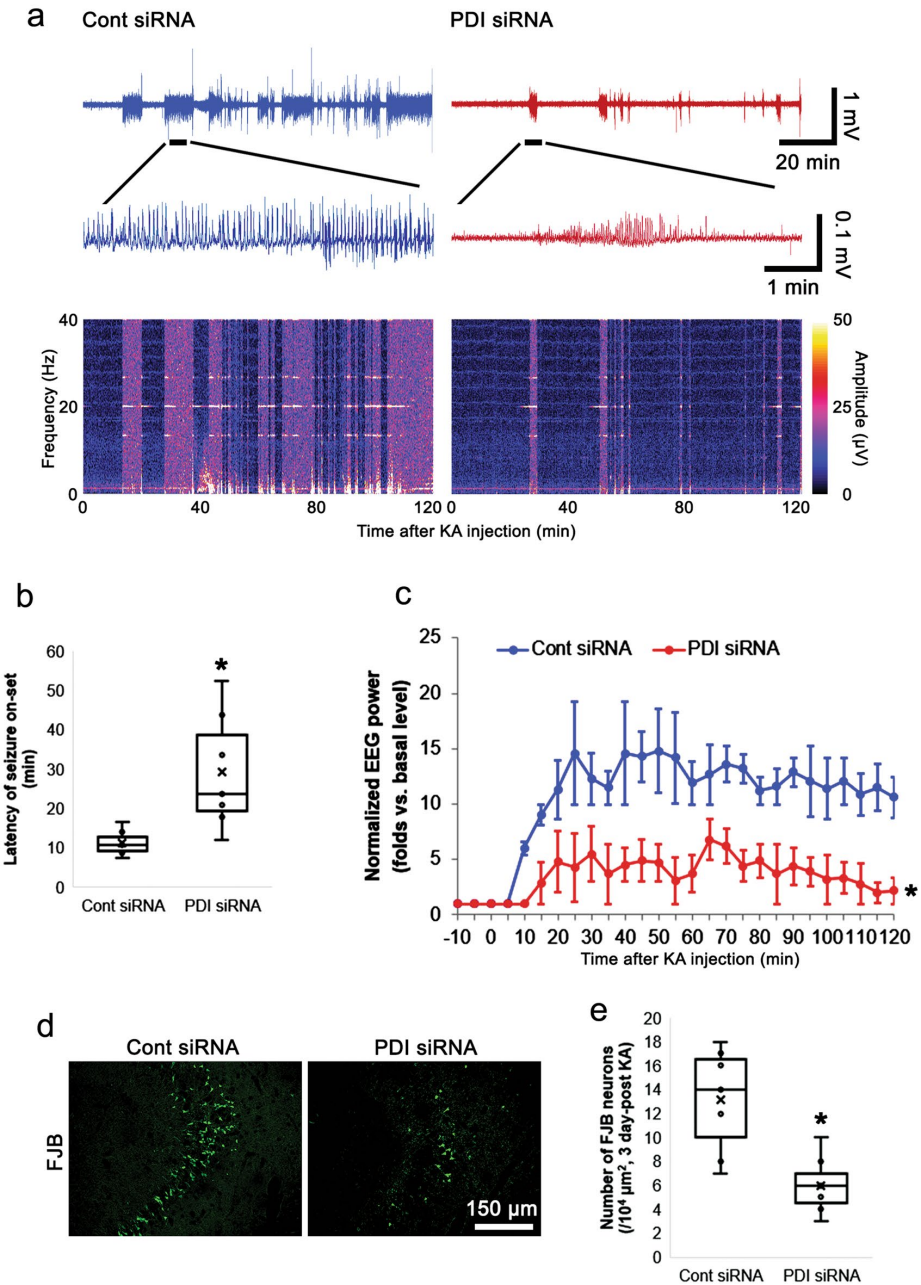
Effects of PDI knockdown on seizure activity and CA3 neuronal damage in response to KA. As compared to control siRNA, PDI siRNA attenuates seizure activity and CA3 neuronal degeneration following KA injection. (**a**) Representative EEG traces (upper panels and middle panels) and frequency-power spectral temporal mans (lower panels) in response to KA. (**b**–**c**) Quantitative analyses of the latency of seiuzre on-set (**b**) and total EEG power (**c**) in response to KA (*p < 0.05 vs. control siRNA; *n* = 7, respectively; Mann–Whitney test and Friedman test). (**d**) Representative images for FJB-positive degenerating CA3 neurons at 3 days after KA injection. (**e**) Quantitation of the number of FJB-positive CA3 neurons (*p < 0.05 vs. control siRNA; *n* = 7, respectively; Mann–Whitney test).

To confirm whether PDI siRNA-induced GluA2 S880 phosphorylation decreases the neuronal activity by enhancing AMPAR internalization, we applied KA (25 mg/kg, i.p.) to animals. This is because AMPAR responses are not desensitized during exposure to KA^22,23^. As compared to control siRNA, PDI siRNA increased the latency of seizure onset in response to KA (Z = 2.875, *p* = 0.004, *n* = 7, respectively, Mann–Whitney test; Fig. 5a–b) and decreased seizure intensity (severity) (χ^2^_(1)_ = 12.532, *p* < 0.001, *n* = 7, respectively, Friedman test; Fig. 5a and c) in response to KA. Compatible with seizure intensity, Fluoro-Jade B (FJB) staining showed that PDI siRNA attenuated KA-induced CA3 neuronal degeneration at 3 days after injection (Z = 2.689, *p* = 0.007, *n* = 7, respectively, Mann–Whitney test; Fig. 5d–e).

### PDI knockdown increases GluA2:PICK1 binding and AMPAR internalization

**Figure 6:**
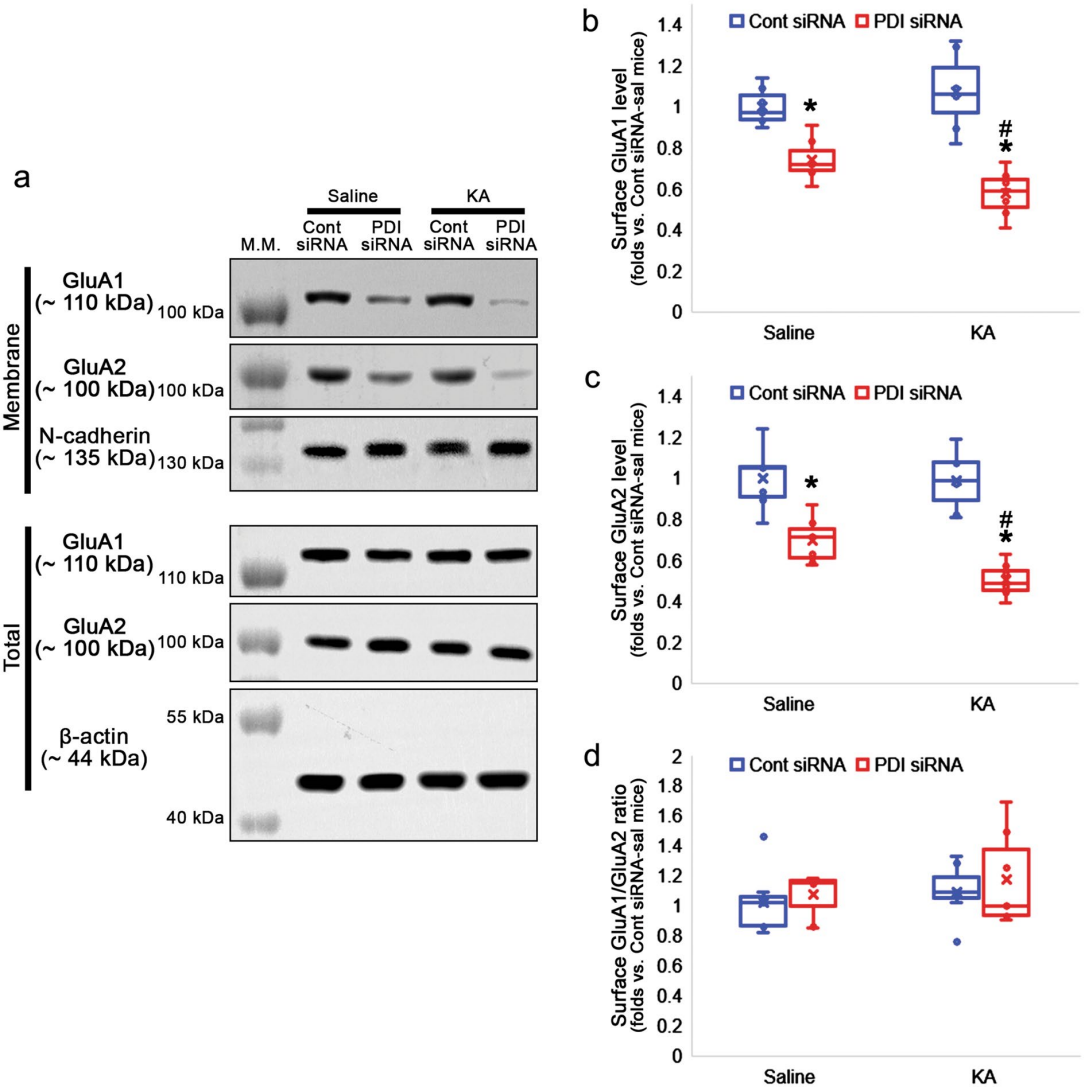
Effects of PDI knockdown on surface AMPAR expression in the hippocampus following KA injection. Control siRNA does not affect surface AMPAR expression in both saline- and KA-treated animals. PDI knockdown decreases surface expression of GluA1 and GluA2 without altering surface GluA1/GluA2 ratio in both saline- and KA-treated animals. PDI knockdown reduces surface AMPAR expression in KA-treated animals more than saline-treated animals. (**a**) Representative Western blot images for surface expressions of GluA1 and GluA2. (**b**–**d**) Quantitative analyses of the effects of PDI siRNA on surface GluA1 (**b**) and GluA2 (**c**) levels and surface GluA1/GluA2 ratio (**d**) following KA injection (*,^#^*p* < 0.05 vs. control siRNA vs. saline; *n* = 7, respectively; Kruskal–Wallis test followed by Tukey *post-hoc* test).

**Figure 7:**
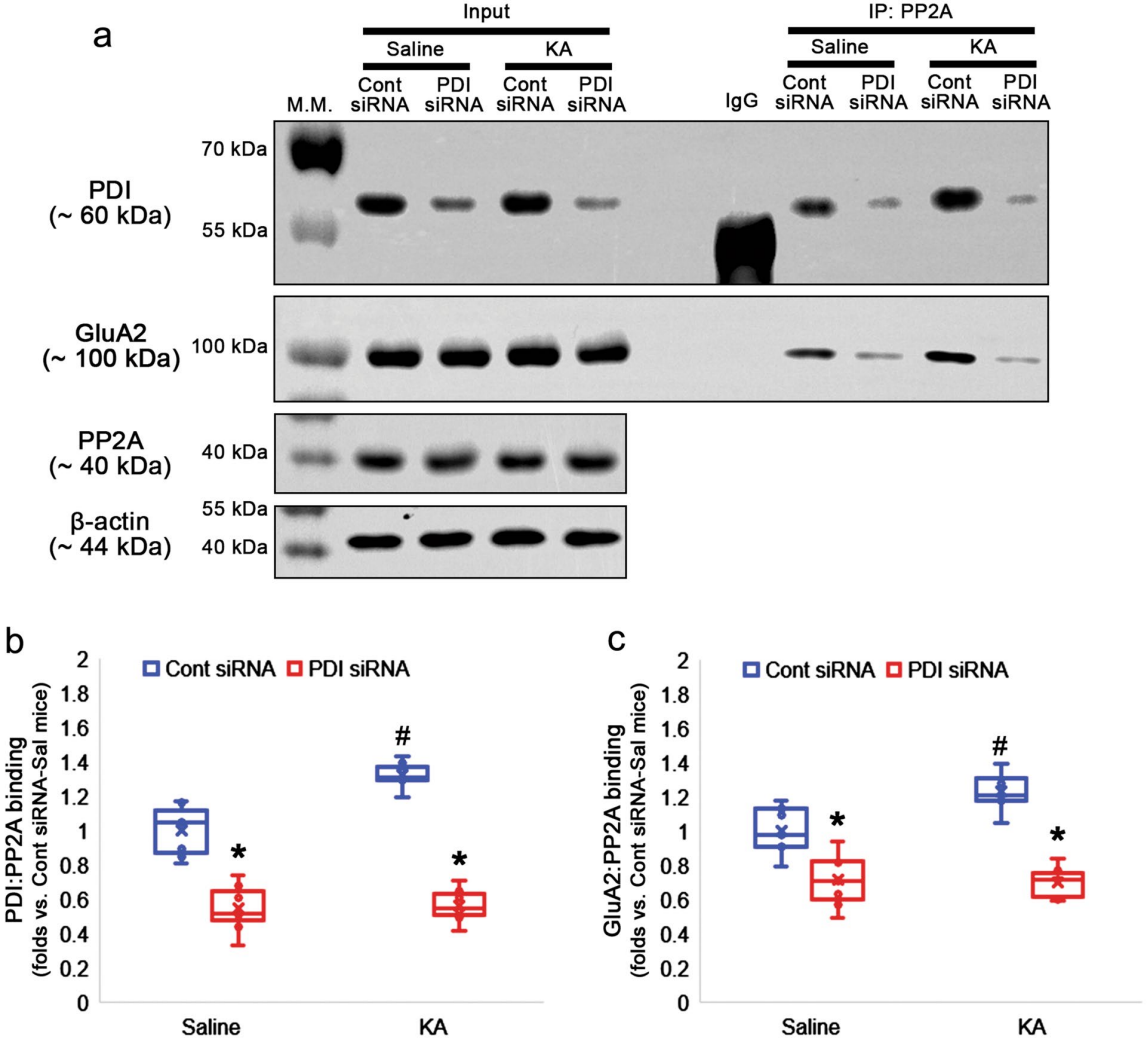
Effects of PDI knockdown on PP2A bindings to PDI and GluA2 in the hippocampus following KA injection. KA increases PDI:PP2A and GluA2:PP2A bindings in control siRNA-infused animals, but not in PDI siRNA-infused animals. (**a**) Representative Western blot images for the PDI:PP2A and GluA2:PP2A bindings. (**b**–**c**) Quantitative analyses of the effects of PDI siRNA on PP2A level (**b**) and PDI:PP2A binding (**c**) following KA injection (*,^#^*p* < 0.05 vs. control siRNA vs. saline; *n* = 7, respectively; Kruskal–Wallis test followed by Tukey *post-hoc* test).

Finally, we evaluated the effect of PDI knockdown on AMPAR internalization in response to KA. KA did not affect surface expressions of GluA1 and GluA2 in control siRNA-treated animals (Fig. 6a–c). As compared to saline, however, KA further reduced surface GluA1 (χ^2^_(3)_ = 20.502, *p* < 0.001, *n* = 7, respectively, Kruskal–Wallis test; Fig. 6a–b, Supplementary Fig. 5) and GluA2 (χ^2^_(3)_ = 21.259, *p* < 0.001, *n* = 7, respectively, Kruskal–Wallis test; Fig. 6a and c, Supplementary Fig. 5) levels in PDI siRNA-treated group. KA did not alter surface GluA1/GluA2 ratio in both control- and PDI siRNA-infused animals (χ^2^_(3)_ = 1.577, *p* = 0.665, *n* = 7, respectively, Kruskal–Wallis test; Fig. 6a and d, Supplementary Fig. 5). Furthermore, KA increased PDI:PP2A binding in control siRNA-infused animals (χ^2^_(3)_ = 22.84, *p* < 0.001, *p* = 0.004, Tukey *post-hoc* test, *n* = 7, respectively, Kruskal–Wallis test; Fig. 7a-b, Supplementary Fig. 6). KA also enhanced GluA2:PP2A binding in control siRNA-infused animals (χ^2^_(3)_ = 22.84, *p* < 0.001, *p* = 0.032, Tukey *post-hoc* test, *n* = 7, respectively, Kruskal–Wallis test; Fig. 7a and c, Supplementary Fig. 6), but not in PDI siRNA-infused animals (*p* = 0.995, Tukey *post-hoc* test; Fig. 7a and b, Supplementary Fig. 6). These findings indicate that PDI-PP2A interaction may prolong seizure activity in response to KA by inhibiting AMPAR internalization.

**Figure 8:**
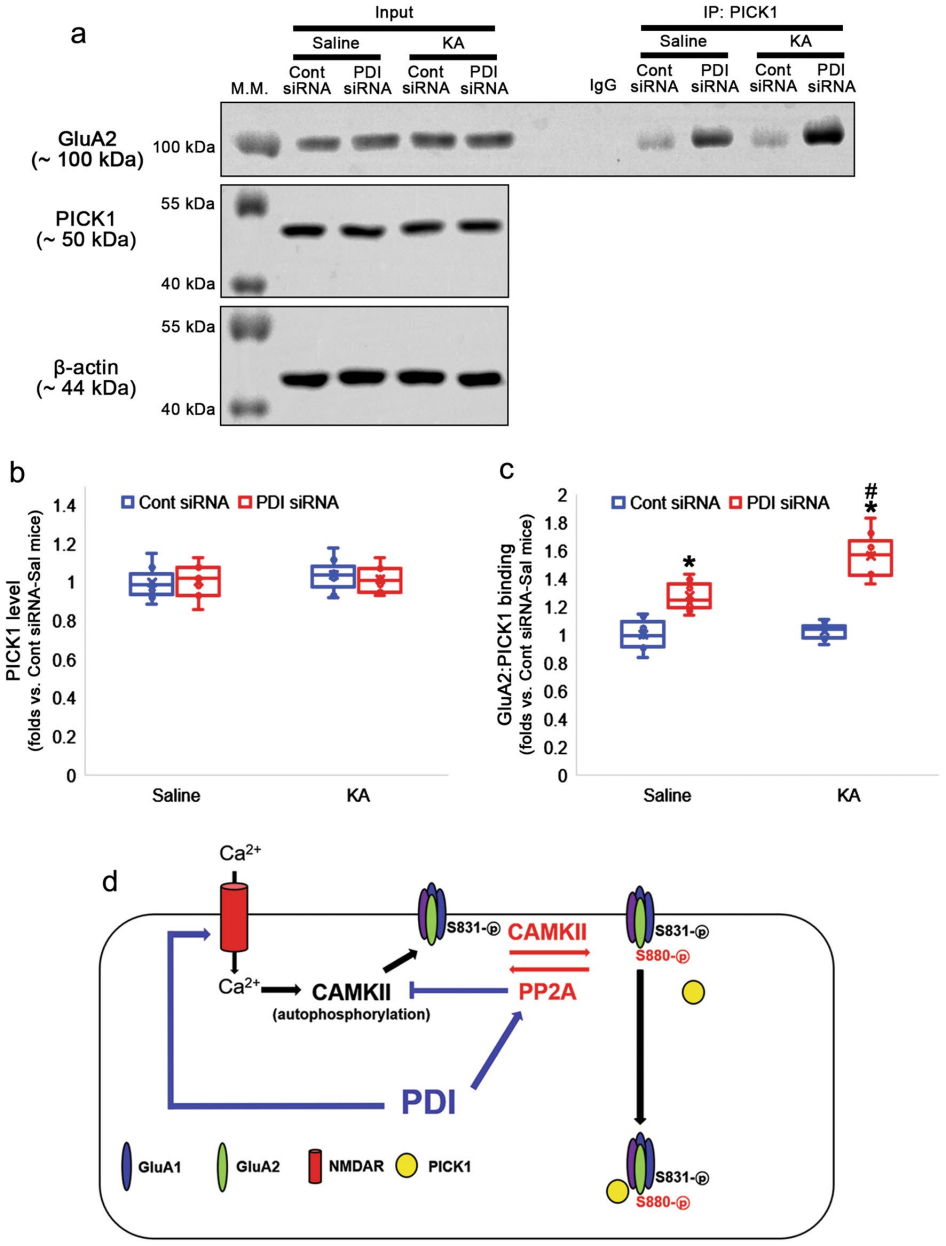
Effects of PDI knockdown on PICK1 bindings to GluA2 in the hippocampus following KA injection. PDI siRNA does not influence PICK1 level in both saline- and KA-treated groups. As compared to control siRNA, PDI siRNA increases GluA2A:PICK1 binding in saline-treated group. Although KA does not affect GluA2:PICK1 binding in control siRNA-infused group, it increases it in PDI siRNA-infused group. (**a**) Representative Western blot images for the GluA2:PICK1 binding. (**b**–**c**) Quantitative analyses of the effects of PDI siRNA on PICK1 level (**b**) and GluA2:PICK1 binding (**c**) following KA injection (*,^#^*p* < 0.05 vs. control siRNA vs. saline; *n* = 7, respectively; Kruskal–Wallis test followed by Tukey *post-hoc* test). (**d**) Scheme of the role of PDI in AMPAR internalization. PDI may reduce lead to reduction-induced PP2A activation, which would abolish PICK1-mediated AMPAR internalization by dephosphorylating GluA2 S880 and CaMKII T286 sites.

GluA2 S880 phosphorylation reinforces its internalization by facilitating GluA2:PICK1 binding, which is regulated by PP2A^35–37^. Therefore, we validated whether PDI knockdown also accelerates GluA2:PICK1 interaction. PDI siRNA did not influence PICK1 level under physiological- and post-KA condition (χ^2^_(3)_ = 0.785, *p* = 0.853, *n* = 7, respectively, Kruskal–Wallis test; Fig. 8a–b, Supplementary Fig. 7). However, PDI siRNA increased GluA2A:PICK1 binding to 1.27-fold of control siRNA level (*p* < 0.001, Tukey *post-hoc* test). KA did not alter GluA2:PICK1 binding in control siRNA-infused animals (*p* = 0.992, Tukey *post-hoc* test), but further enhanced it in PDI siRNA-infused animals (χ^2^_(3)_ = 21.857, *p* < 0.001, *p* = 0.028, Tukey *post-hoc* test, *n* = 7, respectively, Kruskal–Wallis test; Fig. 8a and c, Supplementary Fig. 7). Taken together, our findings indicate that PDI knockdown may lead to oxidation-induced PP2A inhibition, which would facilitate PICK1-mediated AMPAR internalization by increasing GluA2 S880 phosphorylation.

## Discussion

The AMPAR is composed of the homomeric or heteromeric tetramer of four subunits (GluA1, 2, 3 and 4) encoded by different genes^38^. Among them, GluA2 regulates the preference of ion-permeability of AMPAR. GluA2-containing AMPAR activation leads to Na^+^ and K^+^, but not Ca^2+^, influx, while GluA2-lacking AMPAR activation makes Ca^2+^ influx^39^. GluA2-containing AMPAR also shows low channel conductance and a linear rectification pattern, whereas GluA2-lacking AMPAR exhibits high channel conductance and an inward rectification pattern^23^. Thus, GluA2 subunit dominantly inhibits AMPAR-dependent Ca^2+^ permeability as well as channel conductance. Furthermore, GluA2 homotetramer generates steady-state currents when the receptors are desensitized^40^. In the present study, PDI knockdown increased GluA2 S880 phosphorylation concomitant with the reduced surface GluA2 expression. In addition, PDI knockdown enhances GluA1 S831 phosphorylation that increases channel conductance^24,25^. Thus, it is presumable that PDI knockdown would increase the surface expression of Ca^2+^-permeable GluA2-lacing AMPAR or the preponderance of homomeric GluA1 tetramers that would result in much larger single-channel conductance than heteromeric GluA1-GluA2 tetramers through GluA1 S831 phosphorylation^26,27^. However, the present study also shows that PDI siRNA decreased surface GluA1 expression and neuronal activity in response to KA without altering the GluA1/GluA2 ratio. Furthermore, PDI knockdown did not affect GluA1 S845 phosphorylation that drives surface trafficking of GluA1-containing AMPAR and facilitates AMPAR insertion into synaptic membrane to potentiate the peak AMPAR current^11,41,42^. In contrast to S845 phosphorylation, GluA1 S831 phosphorylation is not required for AMPAR trafficking, although it directly enhances the function of AMPAR^26,27^. Given that the GluA1/2 heterotetramer is the most dominant AMPAR subtype in the hippocampal pyramidal cells and > 95% of AMPARs contain the GluA2 subunit under physiological condition^39,43^, our findings indicate that PDI knockdown may diminish neuronal activity in response to KA by reducing surface heteromeric GluA1-GluA2 tetramers through GluA2 S880 phosphorylation without increasing the proportion of homomeric GluR1 tetramers, and that the increased GluA1 S831 phosphorylation may be an adaptive response to the decrease in the total number of AMPAR tetramers.

Surface GluA2 expression is reversely regulated by interactions with glutamate receptor interacting protein 1 (GRIP1) and PICK1. GRIP1 increases surface GluA2 expression, while PICK1 diminishes it^44,45^. GluA2 Y869/Y873/Y876 phosphorylation destabilizes GluA2:GRIP1 interaction and promote its internalization^46,47^. Phosphorylation of GluA2 S880 site also causes dissociation of GRIP1 and subsequently binds to PICK1, then leads to the endocytosis of AMPAR^35,48^. When GluA2 S880 site is dephosphorylated by PP2A, PICK1 dissociates from GluA2 and GRIP1 binds to GluA2^36,37,49^. Thus, the phosphorylation of S880 on GluA2 is an essential step for AMPAR internalization, which is inhibited by Y876 phosphorylation^50^. Compatible with these reports, PDI siRNA increased GluA2 S880 phosphorylation and GluA2:PICK1 binding without affecting GluA2 Y869/Y873/Y876 phosphorylation, which was caused by oxidation-mediated PP2A inhibition under physiological condition, and facilitated GluA2:PICK1 interaction following KA treatment. Regarding that tyrosine phosphorylations of GluA2 is are required for AMPAR internalization^46^ and GluA2-PICK1 interaction is not influenced by tyrosine phosphorylation of GluA2^47^, these findings indicate that PDI knockdown may facilitate GluA2:PICK1 binding and AMPAR internalization independent of tyrosine GluA2 phosphorylation.

Both GluA1 S831 and GluA2 S880 sites are substrate of PKC and CaMKII^12–14^. NMDAR activation initially increases Ca^2+^-dependent PKC activity followed by persistent CaMKII activation^51^. Furthermore, NMDAR-stimulated Ca^2+^ entry activates CaMKII, and subsequent phosphorylates GluA1 at S831 site to increase channel conductance^12,52^. Since PDI enhances NMDAR-mediated neuronal activity through the sulfhydration (reduction) of cysteine residues on the NMDAR redox site^6,10^, it is plausible that PDI siRNA would result in the decrease in GluA1 S831 and GluA2 S880 phosphorylation, accompanied by the reduced PKC T497 and CaMKII T286 phosphorylations (Fig. 8d). However, the present study shows that PDI knockdown reduced PKC T497 phosphorylation, but increased CaMKII T286 phosphorylation concomitant with the enhanced GluA1 S831 and GluA2 S880 phosphorylation. CaMKII is autophosphorylated at T286 site and converted to a Ca^2+^-independent or autonomous species, when Ca^2+^-dependent PKC activation is inhibited^53^. Therefore, our findings suggest that PDI knockdown may lead to CaMKII T286 autophosphorylation, which would increase GluA1 S831 and GluA2 S880 phosphorylations, independent of NMDAR inhibition.

PP1 and PP2A, but not PP2B, dephosphorylates GluA2 S880 site^30^. PP1 and PP2A are also responsible for CaMKII T286 dephosphorylation. In particular, PP2A is the major phosphatase of Ca^2+^-independent T286-autophosphorylated CaMKII^54^. Of interest, PP2A is susceptible to oxidation with an inhibition of phosphatase activity. Indeed, intermolecular disulfide formation (oxidation) between C266 and C269 in PP2A is an inhibitory redox switch in its phosphatase activity^32,33^. Furthermore, PDI has the reductive activity at the cell surface, although it acts predominantly as an oxidase in the ER^55–57^. In the present study, we found that PDI bound to PP2A, but not PP1, and PDI siRNA decreased PDI:PP2A interaction and the amount of free thiols (oxidation) without altering that of SNO-thiols under physiological condition. These findings indicate that PDI downregulation induced by siRNA may decrease the PDI:PP2A binding, which would subsequently diminish the amount of total thiol on PP2A, and suggest that PDI may act as a reductase rather than NO transporter of PP2A. The present data also show that KA increased PDI:PP2A and GluA2:PP2A bindings without altering surface GluA1/2 expression and GluA2:PICK1 interaction in control siRNA-infused animals. Although we could not provide the underlying mechanisms in the present study, it is likely that these phenomena may be maladaptive responses to KA-induced seizures: KA may augment PDI-mediated sulfhydration (reduction) of PP2A and the subsequent PP2A-mediated GluA2 S880 dephosphorylation, which would prolong seizure activity in response to KA. Since GluA2 S880 phosphorylation facilitates AMPAR internalization, in turn, PP2A-mediated GluA2 S880 dephosphorylation may result in the unchanged GluA2 surface expression upon KA treatment by abrogating PICK1-mediated AMPAR internalization. These are compatible with previous studies demonstrating that KA is a non-desensitizing agonist of AMPAR^22,23^. In contrast, PDI siRNA attenuated seizure activity in response to KA and further decreased surface GluA1/2 expression concomitant with augmented GluA2:PICK1 interaction following KA injection as compared to saline treatment, although it did not change GluA2:PP2A binding. Regarding that KA activates PP2A and decreases CaMKII activity and its autophosphorylation in the mouse hippocampus at 2.5–6 h after injection^58,59^, these findings also indicate that PDI may abolish AMPAR internalization by activating PP2A. Given that the clear loss of PP2A activity correlates with the cysteine oxidation^60^, our findings suggest that PDI siRNA may increase GluA1 S831, GluA2A S880 and CaMKII T286 phosphorylations by inducing oxidation-mediated PP2A inhibition under physiological condition, which would facilitate PICK1-mediated AMPAR internalization following KA treatment.

On the other hand, KA shows a biphasic effect on presynaptic transmission in the hippocampus and other brain regions. Low KA concentrations (50–100 nM) increase glutamate release, while higher concentrations decrease it^61^. In the amygdala, KA receptor (KAR)-activation induces a depression of NMDA and AMPA-mediated evoked excitatory postsynaptic current (eEPSC) amplitude, which is prevented by PKA, but not PKC, inhibitors^62^. In the hippocampus, KAR activation also negatively modulates glutamate release by convergence with group II metabotropic glutamate receptors (mGluRII, inhibitory autoreceptors in presynaptic region) through PKA^63,64^. In contrast, KAR activation facilitates glutamate release through Ca^2+^-CAM-mediated PKA activation in the hippocampus and thalamocortical synapses^65,66^. Thus, it is presumable that PDI would regulate seizure susceptibility to KA by affecting presynaptic KAR. However, the present study shows that PDI knockdown inhibited PKC, but increased CaMKII activity without affecting PKA activity under physiological condition. Considering that activation of PKA, but not PKC, is required for KAR-mediated regulation of presynaptic glutamate release^62,63,65,66^, our findings suggest that PDI may not influence presynaptic glutamate release in response to KA.

In conclusion, the present data indicate that PDI played a crucial role in PP2A activation by reducing disulfide bonds, which abolished AMPAR internalization by dephosphorylating GluA2 S880 and CaMKII T286 sites and abrogating GluA2:PICK1 interaction (Fig. 8d). Therefore, our findings provide a novel mechanism to modify AMPAR-mediated responses under physiological and pathological conditions.

## Methods

### Ethics and guidelines

All experimental animal protocols were approved by the Animal Care and Use Committee of Hallym University (#Hallym 2021-30, approval date: May 17, 2021). This study was also carried out in compliance with the ARRIVE guidelines.

### Experimental animals and chemicals

Male C57BL/6J mice (8 weeks old) were used in the present study. Animals were provided with a commercial diet and water ad libitum under controlled temperature, humidity and lighting conditions (22 ± 2 °C, 55 ± 5% and a 12:12 light/dark cycle). All reagents were obtained from Sigma-Aldrich (USA), except as noted.

### Surgery and PDI knockdown

Mice were anesthetized with Isoflurane (3% induction, 1.5–2% for surgery and 1.5% maintenance in a 65:35 mixture of N_2_O:O_2_). A brain infusion kit 3 (Alzet, USA) was implanted into the right lateral ventricle (1 mm posterior; 1.5 mm lateral; 3.5 mm depth from bregma) and connected to an osmotic pump (1007D, Alzet, USA) containing a non-silencing RNA (control siRNA) or PDI siRNA (CCUUUGCUAGCGAAUCUCAGAGCC), which were continuously infused over 6-day period. Some animals were also implanted monopolar electrode (Plastics One, USA) into the left dorsal hippocampus (2 mm posterior; 1.25 mm lateral; 2 mm depth from bregma)^6,10,18,19,21^.

### Seizure induction and electroencephalogram recording

Three days after surgery, mice were given KA (25 mg/kg, i.p.) or an equal volume of normal saline instead of KA. Saline-treated mice were used as controls. Diazepam (Valium; Roche, France; 10 mg/kg, i.p.) was administered 2 h after KA injection. Electrode-implanted animals were also given KA (25 mg/kg, i.p.) after baseline recording for at least 30 min. Electroencephalographic (EEG) signals were recorded with a DAM 80 differential amplifier (0.1–1000 Hz bandpass; World Precision Instruments, USA) and digitized (1000 Hz) using LabChart Pro v7 software (AD Instruments, Australia). EEG total power was obtained from integration of EEG amplitude in the frequency bands from 0 to 80 Hz. Frequency-power spectral temporal maps were generated with filtrations of the frequency domain (0–80 Hz) and the amplitude domain (0–50 mV). Total power was measured during the 2 h recording session and spectrograms were automatically calculated using a Hanning sliding window with 50% overlap by LabChart Pro v7 software (AD Instruments, Australia). Total EEG power was normalized by the baseline power obtained from each animal. Latency of seizure on-set was defined as the time point showing more than 3 s and consisting of a rhythmic discharge between 4 and 10 Hz with amplitude of at least two times higher than the baseline EEG. After recording, animals were used for biochemical experiments^6,10,18,19,21^.

### Western blot

Animals were sacrificed by decapitation under urethane anesthesia (1.5 g/kg, i.p.). The hippocampus was dissected out and homogenized in lysis buffer (50 mM Tris containing 50 mM 4-(2-hydroxyethyl)-1-piperazineethanesulfonic acid (pH 7.4), ethylene glycol tetraacetic acid (pH 8.0), 0.2% Tergitol type NP-40, 10 mM ethylenediaminetetraacetic acid (pH 8.0), 15 mM sodium pyrophosphate, 100 mM β-glycerophosphate, 50 mM NaF, 150 mM NaCl, 2 mM sodium orthovanadate, 1 mM phenylmethylsulfonyl fluoride, and 1 mM dithiothreitol). Total protein content was measured by BCA protein assay kit. Western blot was performed according to standard procedures (Table 1). The signals were scanned and analyzed by ImageQuant LAS4000 system (GE health). The values of each sample were normalized with the corresponding amount of anti-β-actin (input) or N-cadherin (membrane fraction). The ratio of phosphoprotein to total protein was described as phosphorylation levels^6,10,18,19,21^.

**Table 1:** Primary antibodies used in the present study.

Antigen	Host	Manufacturer (catalog number)	Dilution used
CaMKII	Rabbit	Santa Cruz (sc-13082)	1:1,000 (WB)
GRIA1	Rabbit	Synaptic Systems (182011)	1:1000 (WB)
GRIA2	Rabbit	Sigma (AB1768-I)	1:1,000 (WB)
N-cadherin	Rabbit	Abcam (ab18203)	1:4000 (WB)
NeuN	Guinea pig	Millipore (#ABN90P)	1:1000 (IH)
p-CaMKII T286	Rabbit	Abcam (ab32678)	1:2500 (IH)
p-GRIA1 S831	Rabbit	Abcam (ab109464)	1:1000 (WB)
p-GRIA1 S845	Rabbit	Millipore (#AB5849)	1:1000 (WB)
p-GRIA2 S880	Rabbit	Invitrogen (#PA5-38134)	1:100 (IH) 1:1000 (WB)
p-GRIA2 Y869/873/876	Rabbit	Cell signaling (#3921)	1:1,000 (WB)
p-PKA T197	Rabbit	Assay Biotech (A0548)	1:1000 (WB)
p-PKC (catalytic subunit) T497	Rabbit	Abcam (ab76016)	1:1000 (WB)
PDI	Rabbit	Cell signaling (#2446)	1:1000 (WB)
PICK1	Rabbit	Abcam (ab3420)	1:50 (IP) 1:1000 (WB)
PKA (catalytic subunit)	Rabbit	BioVision (3115-100)	1:1000 (WB)
PKC	Rabbit	Abcam (ab23511)	1:1000 (WB)
PP1	Rabbit	Abcam (ab52619)	1:100 (IP) 1:1000 (WB)
PP2A	Rabbit	Cell signaling (#2038)	1:100 (IP) 1:1000 (WB)
β-actin	Mouse	Sigma (A5316)	1:5000 (WB)

*IH* immunohistochemistry, *IP* immunoprecipitation, *WB* western blot.

### Co-immunoprecipitation and membrane fraction

The tissues were lysed in radioimmune precipitation buffer (RIPA) with protease and phosphatase inhibitor cocktails (Roche Applied Sciences) and 1 mM sodium orthovanadate. After quantification of total protein concentration, equal amounts of protein (amount in 150 μg) were precipitated with the PDI, PP1, PP2A or PICK1 antibody and subsequently incubated with protein G sepharose at 4 °C overnight. Beads were collected, eluted in sample buffer and boiled at 95 °C for 5 min. The elution step was performed under the same conditions and in parallel. To analyze membrane expressions of GluA1 and GluA2, subcellular Protein Fractionation Kit for Tissues (Thermo Scientific, USA) was used according to the manufacturer’s instructions. Next, Western blot was performed according to standard procedures^6,10,18,19,21^.

### Measurement of SNO-thiol- and total thiol on PP2A

Modified biotin switch assay was performed with the *S*-nitrosylation Western Blot Kit (ThermoFisher, USA) according to the manufacturer's protocol. Briefly, lysates were reacted with ascorbate in HENS buffer for specific labeling with iodoTMTzero reagents with MMT pretreatment (for SNO-thiol), or not (for total thiol). Protein labeling can be confirmed by Western blot using TMT antibody. Thereafter, TMT-labeled proteins were purified by Anti-TMT Resin, eluted by TMT elusion buffer, and identified by Western blot according to standard procedures. For technical controls, we omitted ascorbate for each sample. The ratio of SNO-protein or total thiol-protein to total protein was described as *S*-nitrosylation or total thiol levels^6,10,18,19,21^.

### Immunohistochemistry and FJB staining

Animals were anesthetized with urethane anesthesia (1.5 g/kg, i.p.) and perfused transcardially with 4% paraformaldehyde in 0.1 M phosphate buffer (PB, pH 7.4). Brains were post-fixed in the same fixative overnight and then cryoprotected and sectioned at 30 μm with a cryostat. Free-floating coronal sections were incubated in PDI antibody in PBS containing 0.3% Triton X-100 overnight at room temperature. Tissue sections were incubated with a mixture of guinea pig anti-NeuN and rabbit p-GluA2 S880 antibodies in PBS containing 0.3% Triton X-100 overnight at room temperature. Thereafter, sections were visualized with Cy2- and Cy3-conjugated secondary antibodies. Immunoreaction was observed and analyzed using an AxioImage M2 microscope (Carl Zeiss, Germany). To establish the specificity of the immunostaining, a negative control test was carried out with preimmune serum instead of the primary antibody. No immunoreactivity was observed for the negative control in any structures. All experimental procedures in this study were performed under the same conditions and in parallel. To analyze the neuronal damage, we also applied FJB staining. Three days after KA treatment, sections were prepared as the same methods aforementioned. Sections were placed on slides, dried, and immersed in 80% ethanol containing 1% sodium hydroxide. Next, slides were immersed in 70% ethanol solution for 2 min and in purified water for 2 min. After immersion in 0.06% potassium permanganate solution for another 10 min, slides were rinsed with purified water for 2 min. Then, slides were were incubated for 30 min in 0.001% FJB (Histo-Chem Inc., USA), freshly prepared by adding 20 ml of a 0.01% stock FJB solution to 180 ml of 0.1% acetic acid, with gentle shaking in the dark. After mounting, areas of interest (the CA3 pyramidal cell layer, 10^4^ μm^2^) were captured, and were selected. Thereafter, cells count was performed using AxioVision Rel. 4.8 software^6,10,18,19,21^.

### Statistical analysis

Comparisons of data among groups were analyzed by Mann–Whitney test, Friedman test or Kruskal–Wallis test followed by Tukey *post-hoc* test. A *p* < 0.05 is considered to be statistically different.

### Supplementary Information

Supplementary Information.

## Data Availability

The datasets used and/or analysed during the current study available from the corresponding author on reasonable request.
