# Supplementary material for: PDI augments kainic acid-induced seizure activity and neuronal death by inhibiting PP2A-GluA2-PICK1-mediated AMPA receptor internalization in the mouse hippocampus
Source: Sci Rep. 2023 Aug 25;13:13927. doi: 10.1038/s41598-023-41014-7 (PMC10457386; doi:10.1038/s41598-023-41014-7)
Supplement: Supplementary file 1 — Supplementary Information. [file 41598_2023_41014_MOESM1_ESM.pdf]

Supplementary Information

PDI augments kainic acid-induced seizure activity and neuronal death by inhibiting PP2A-GluA2-PICK1-mediated AMPA receptor internalization in the mouse hippocampus

Duk-Shin Lee[1,2], Tae-Hyun Kim[1,2] , Hana Park[1,2] and Ji-Eun Kim[1,2]*

[1] Department of Anatomy and Neurobiology, College of Medicine, Hallym University, Chuncheon 24252, South Korea

[2] Institute of Epilepsy Research, College of Medicine, Hallym University, Chuncheon 24252, South Korea

* Correspondence to: J. -E. Kim, Department of Anatomy and Neurobiology, College of Medicine, Hallym University, Chuncheon, Kangwon-Do 24252, South Korea; Tel: +82-33-248-2522; Fax: +82-33-248-2525; E-mail: jieunkim@hallym.ac.kr

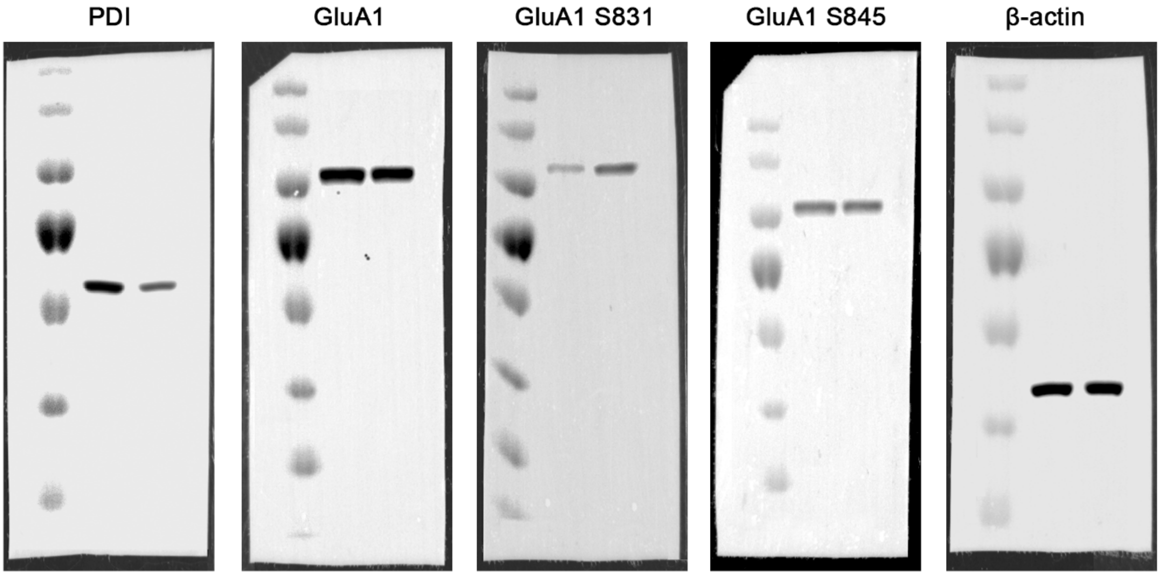

Supplementary Figure 1. Full-length gel images of Western blots in Figure 1.

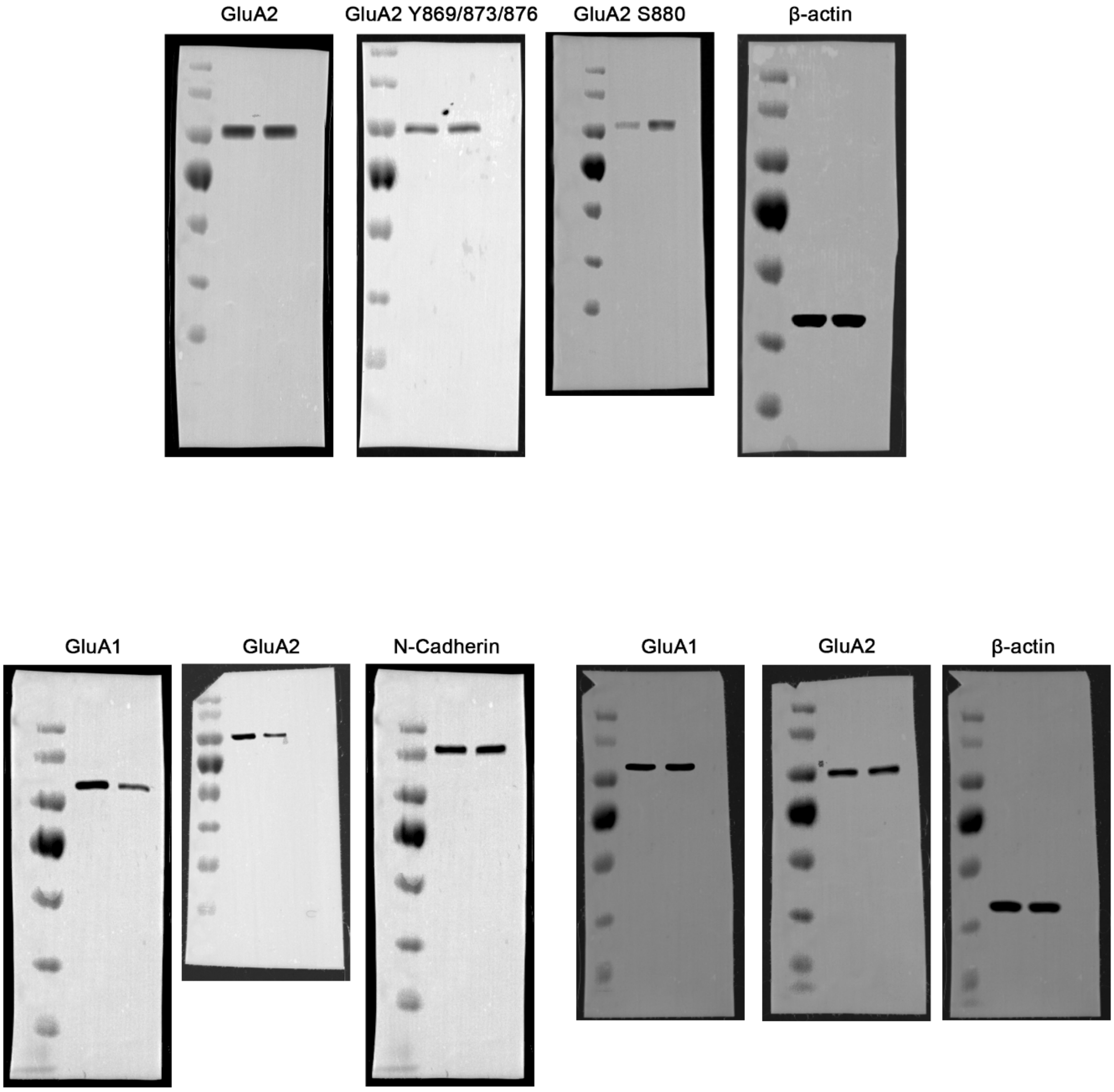

Supplementary Figure 2. Full-length gel images of Western blots in Figure 2.

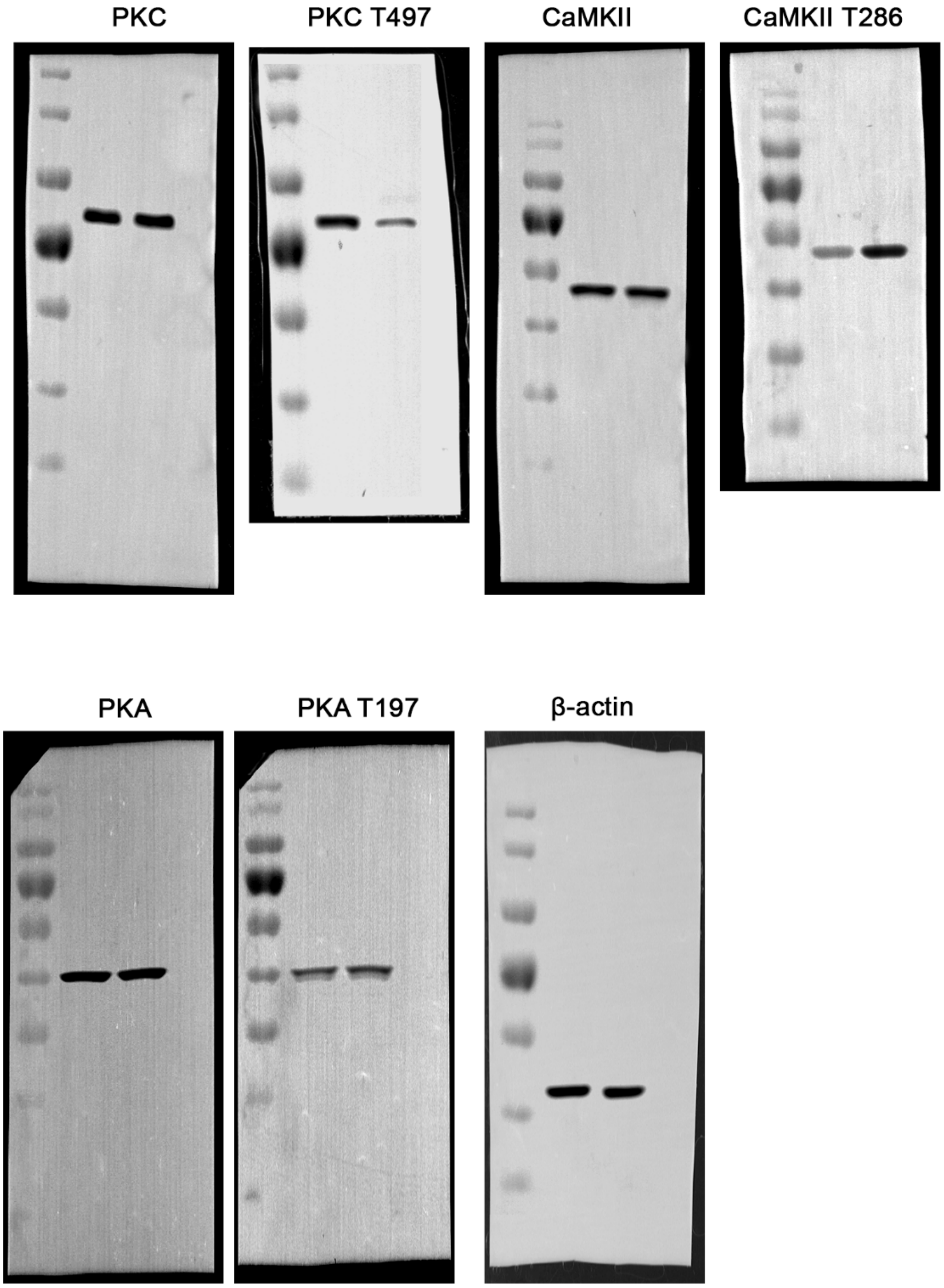

Supplementary Figure 3. Full-length gel images of Western blots in Figure 3.

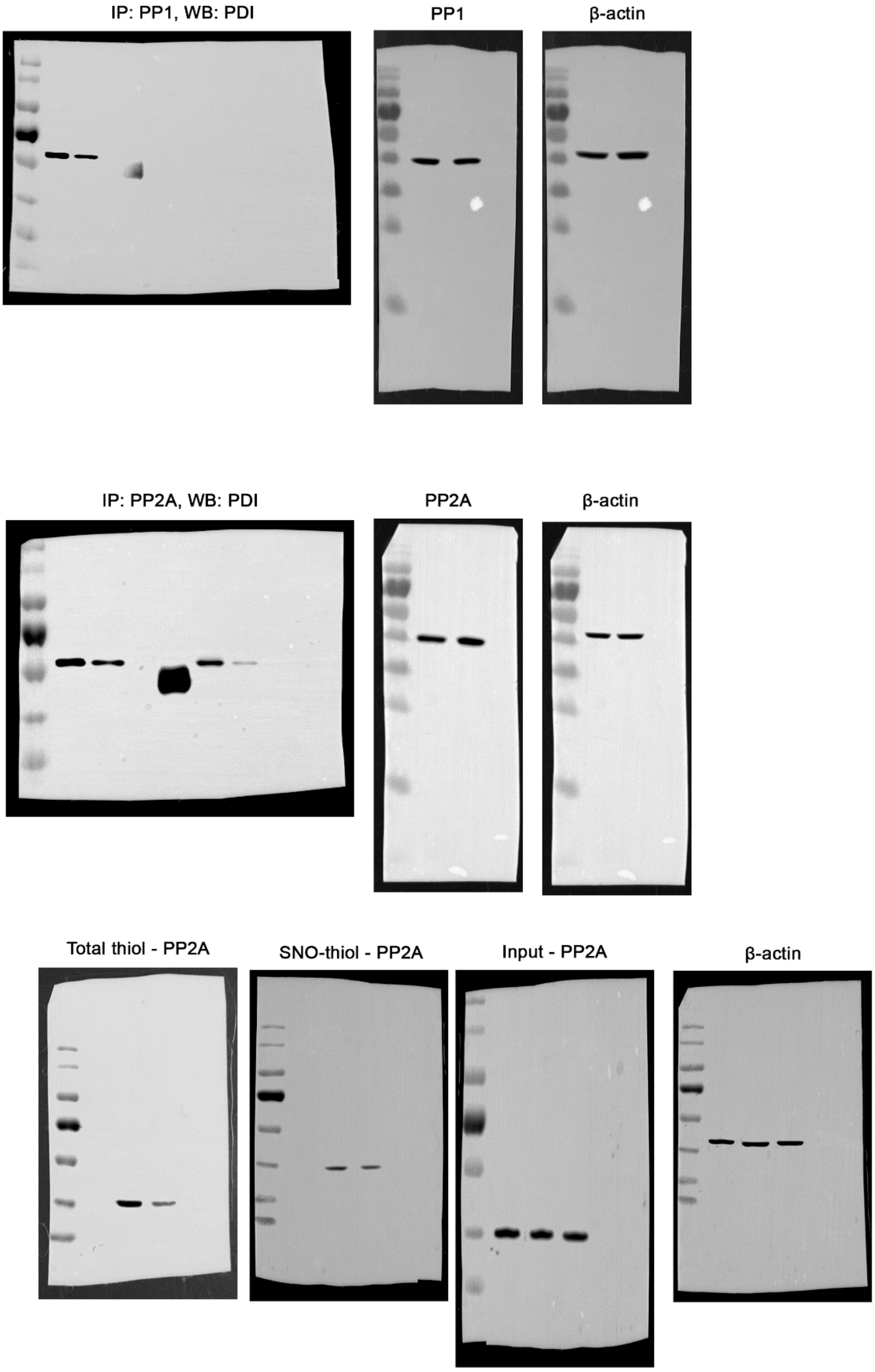

Supplementary Figure 4. Full-length gel images of Western blots in Figure 4.

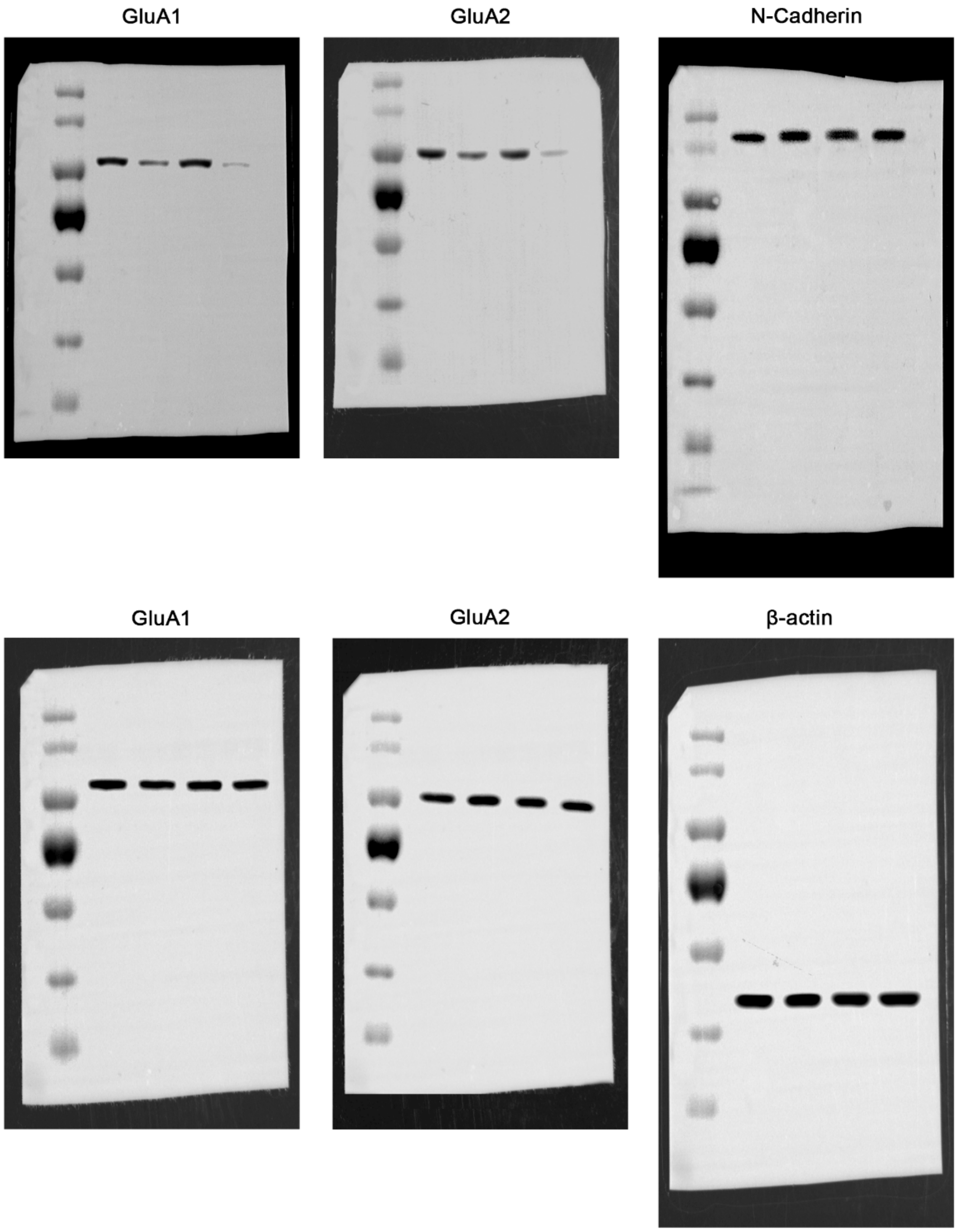

Supplementary Figure 5. Full-length gel images of Western blots in Figure 6.

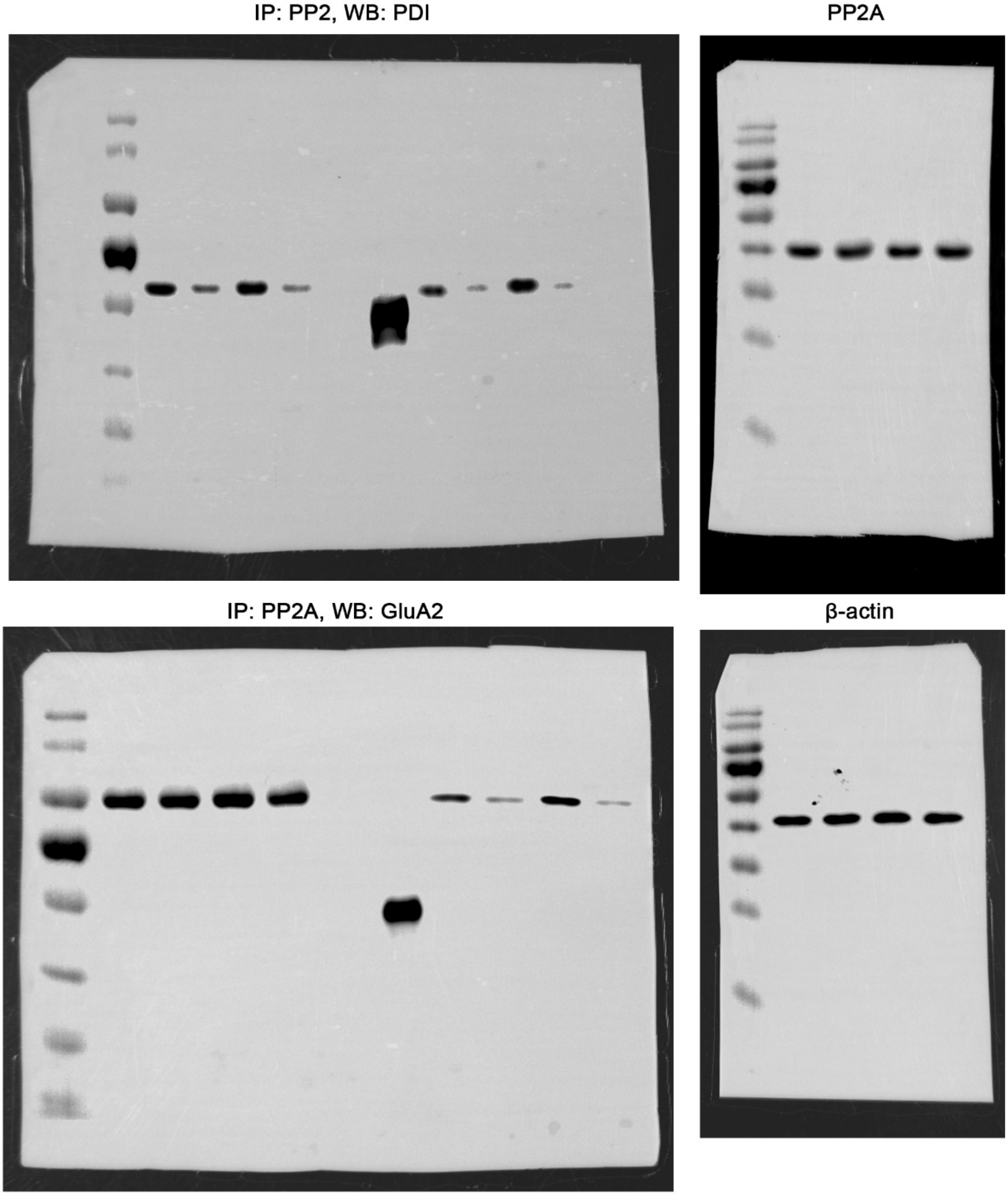

Supplementary Figure 6. Full-length gel images of Western blots in Figure 7.

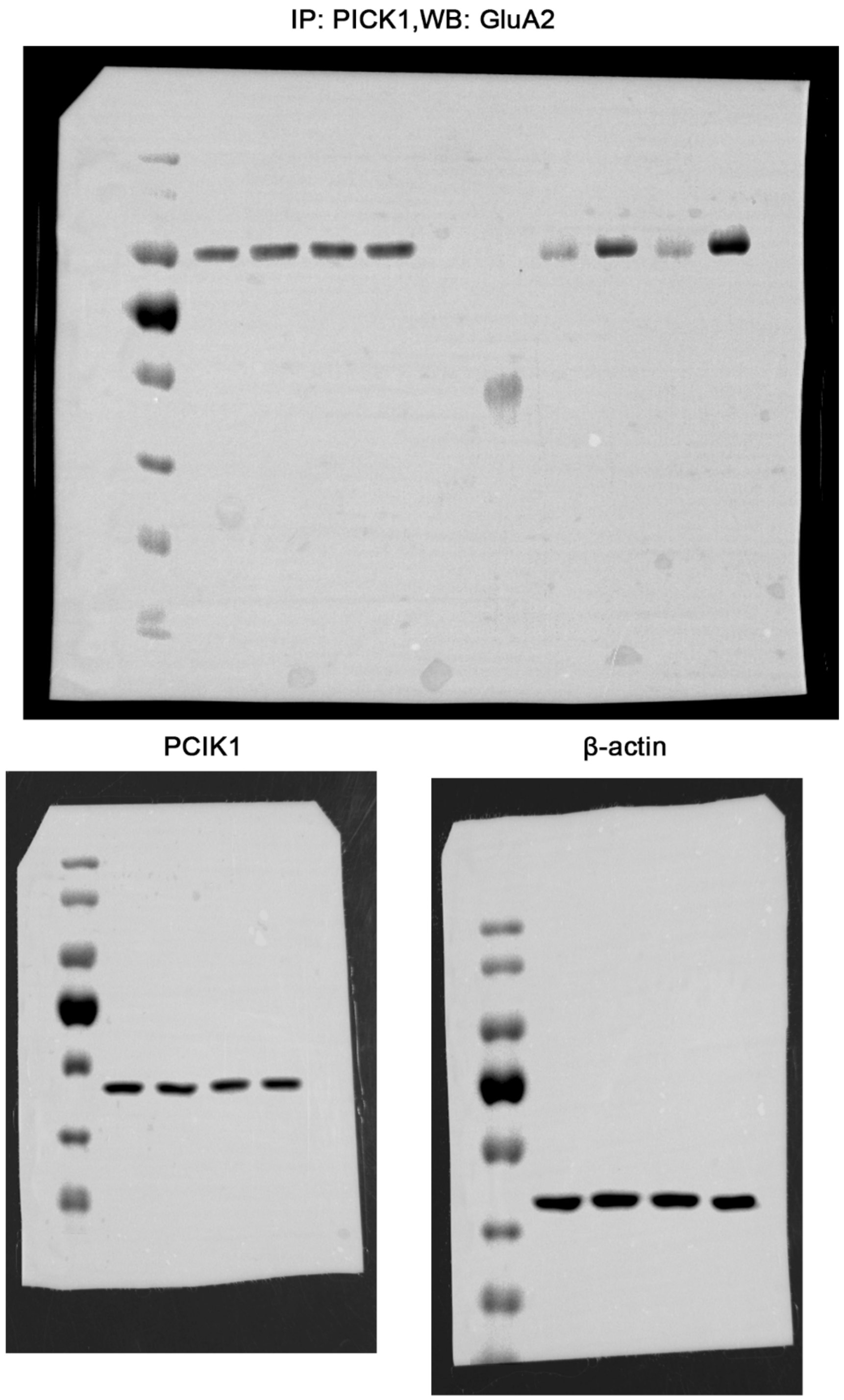

Supplementary Figure 7. Full-length gel images of Western blots in Figure 8.
